# Transition-metal-catalyzed C–H bond activation as a sustainable strategy for the synthesis of fluorinated molecules: an overview

**DOI:** 10.3762/bjoc.19.35

**Published:** 2023-04-17

**Authors:** Louis Monsigny, Floriane Doche, Tatiana Besset

**Affiliations:** 1 Normandie University, INSA Rouen, UNIROUEN, CNRS, COBRA (UMR 6014), 76000 Rouen, France

**Keywords:** C–H bond activation, emergent fluorinated groups, homogeneous catalysis, organofluorine chemistry, palladium, synthetic method

## Abstract

The last decade has witnessed the emergence of innovative synthetic tools for the synthesis of fluorinated molecules. Among these approaches, the transition-metal-catalyzed functionalization of various scaffolds with a panel of fluorinated groups (XR_F_, X = S, Se, O) offered straightforward access to high value-added compounds. This review will highlight the main advances made in the field with the transition-metal-catalyzed functionalization of C(sp^2^) and C(sp^3^) centers with SCF_3_, SeCF_3_, or OCH_2_CF_3_ groups among others, by C–H bond activation. The scope and limitations of these transformations are discussed in this review.

## Introduction

Nowadays, fluorinated molecules represent an indispensable class of molecules in chemistry and in chemical biology. Thanks to the unique properties of the fluorine atom or fluorinated groups to modulate biological and physicochemical properties of the parent non-fluorinated molecules [1–2], their incorporation in various scaffolds afforded high value-added compounds as demonstrated by their numerous applications in the industrial sector such as drugs, agrochemicals, and materials. To further illustrate the ubiquity of fluorinated compounds, almost 20% of pharmaceuticals and 30–40% of agrochemicals [3–5] contain at least one fluorine atom. Because of the exceptional features of fluorinated derivatives, tremendous developments and discoveries have been made in this blossoming research area, with a high interest for fundamental research as well as the industry [6–11]. Among them [2,5,12–18], the direct functionalization of a simple C–H bond by transition-metal catalysis [19–43] became an important tool offering new retrosynthetic disconnections. In this context, a strong interest from the scientific community was shown towards the challenging synthesis of fluorinated molecules by transition-metal-catalyzed C–H bond activation [44–50], allowing the functionalization of complex molecules and even for late-stage functionalization strategy [51–53] for the synthesis of natural products [42,54–59].

**Scheme 1:**
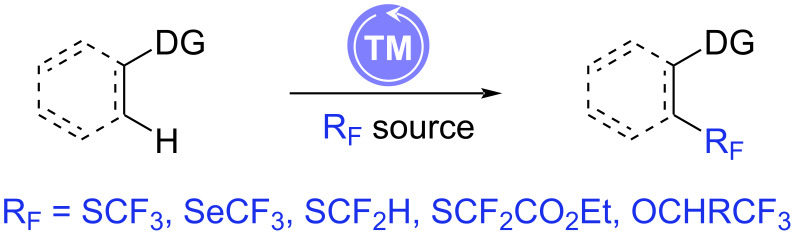
Transition-metal-catalyzed C–XR_F_ bond formation by C–H bond activation: an overview.

The goal of this review is to highlight and discuss the recent approaches for the synthesis of fluorinated derivatives by the direct incorporation of a fluorinated group of XR_F_ type (e.g., SCF_3_, SeCF_3_, SCF_2_CO_2_Et, OCH_2_CF_3_) by transition-metal-catalyzed C–H bond activation (Scheme 1). The review will be organized in two main parts, dedicated to the construction of a C–SCF_2_R/SeCF_3_ and C–OCH_2_CF_3_ bond. This review does not aim to be exhaustive and key examples were carefully chosen to provide the reader a nice overview. Since reviews dealing with transition-metal-catalyzed functionalization of compounds by C–H bond activation with fluorinated reagents [60–77] and vinylic, allylic or propargylic fluorinated building blocks [49] have been already reported, these reactions will not be included.

## Review

### I. Transition-metal-catalyzed directed C–chalcogen bond formation (C–S, C–Se) by C–H bond activation

In the past decade, particular attention has been paid to the development of new methodologies for the incorporation of sulfur-containing fluorinated groups. Although the transition-metal-catalyzed direct C–H bond functionalization appeared to be a powerful tool for the installation of C–C, C–N, or C–O bonds, the direct formation of a C(sp^2^)–SR_F_ or a C(sp^3^)–SR_F_ bond remains a challenging task. In this context, key players in the field have been interested in the design of original methodologies for the trifluoromethylthiolation and more recently the difluoromethylthiolation of various compounds by transition-metal catalysis [78]. Moreover, a recent interest was devoted to the trifluoromethylselenolation reaction as depicted in this section.

#### I.1) Transition-metal-catalyzed C–H trifluoromethylthiolation of aromatic C(sp^2^) centers

Thanks to its unique features such as an interesting lipophilicity (Hansch parameter = 1.44) [79–80] and a strong withdrawing character, the development of new methodologies for the incorporation of the SCF_3_ residue on various molecules has known a tremendous expansion [81–113].

**Scheme 2:**
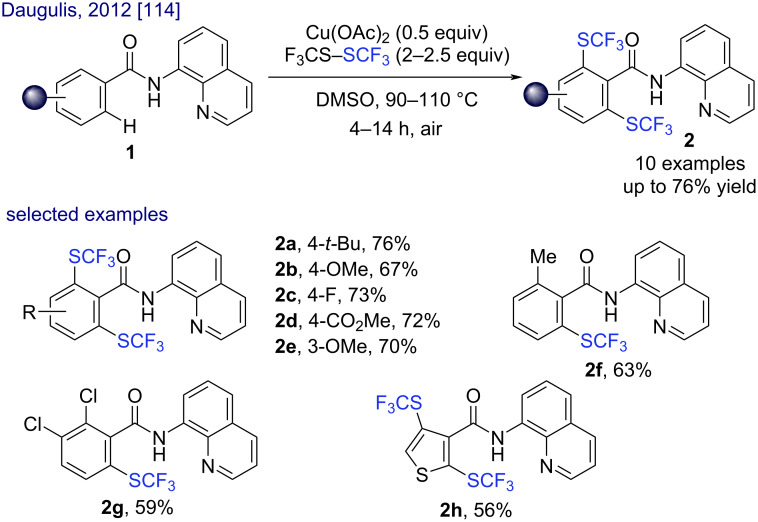
Cu(OAc)_2_-promoted mono- and ditrifluoromethylthiolation of benzamide derivatives derived from 8-aminoquinoline reported by the group of Daugulis [114].

**Copper catalysis:** In 2012, Daugulis and co-workers reported the copper-promoted trifluoromethylthiolation of benzamide derivatives **1** at the *ortho*-position by C–H bond activation [114]. Indeed, using a bidentate directing group (amide derived from the 8-aminoquinoline), the mono- and difunctionalized compounds were obtained when Cu(OAc)_2_ (0.5 equiv) and the toxic and volatile disulfide F_3_CS**–**SCF_3_ were employed (Scheme 2, 10 examples, up to 76% yield). With this approach, derivatives bearing an aromatic part substituted at the *para*-position with electron-donating substituents (**1a**,**b**), halogens (**1c**) as well as electron-withdrawing groups (**1d**) were difunctionalized in good yields. The substitution pattern on the aromatic ring did not affect the reaction efficiency, the *meta*-substituted derivative **2e** as well as the *ortho*-substituted derivative **2f** were obtained in high yields (70% and 63% yields, respectively). It should be noted that the presence of *ortho*-substituents on the aryl residue allowed the monofunctionalization to occur selectively. Also, amide **1g** bearing a disubstituted arene was successfully functionalized in 59% yield. Finally, the difunctionalized thiophene derivative **2h** was obtained in 56% yield.

**Scheme 3:**
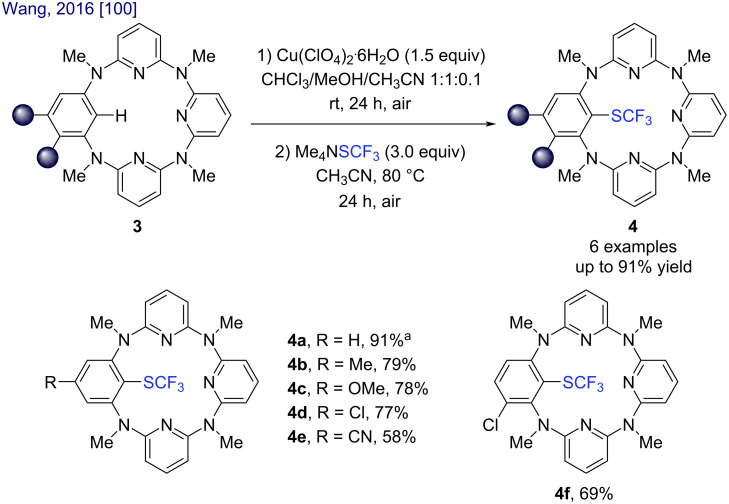
Trifluoromethylthiolation of azacalix[1]arene[3]pyridines using copper salts and a nucleophilic SCF_3_ source reported by Wang and co-workers [100]. ^a^A mixture of CHCl_3_/MeOH 1:1 was used as solvent.

In 2016, Wang's group developed another methodology for the trifluoromethylthiolation of azacalix[1]arene[3]pyridines by C–H bond activation using a complex of Cu(ClO_4_)_2_·6H_2_O and the shelf-stable Me_4_NSCF_3_ [115–116] as a nucleophilic source of SCF_3_ (Scheme 3) [100]. Within these conditions, a set of six azacalix[1]arene[3]pyridines bearing electron-donating groups, halogens or electron-withdrawing groups were functionalized and the expected products were isolated in moderate to high yields (**4a**–**f**, 58–91%).

**Scheme 4:**
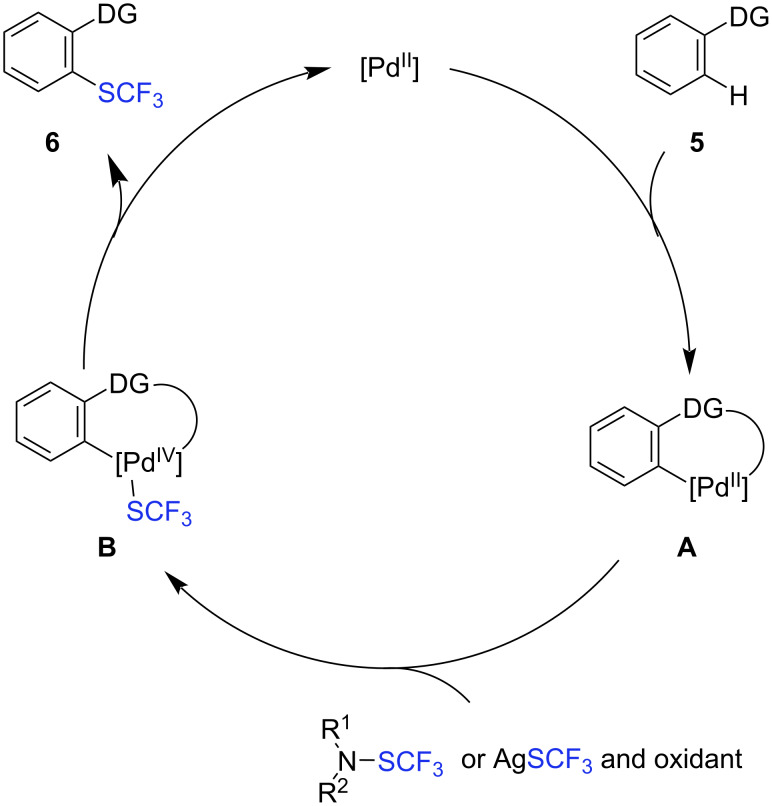
Working hypothesis for the palladium-catalyzed C–H trifluoromethylthiolation reaction.

**Palladium catalysis:** Several works have been reported for the palladium-catalyzed trifluoromethylthiolation reaction of various aromatic compounds **5** by C–H bond activation and involved in most cases an electrophilic SCF_3_ source (R^1^R^2^NSCF_3_). For these transformations, the following working hypothesis was generally suggested (Scheme 4). After coordination of the palladium catalyst to a directing group, the metallacycle **A** is formed. This latter undergoes an oxidative addition in the presence of an electrophilic source or an oxidation/ligand exchange in the presence of a nucleophilic source (i.e., AgSCF_3_) and an oxidant (**B** in Scheme 4). Finally, after a reductive elimination step, the expected functionalized product **6** is obtained and the palladium catalyst is regenerated.

**Scheme 5:**
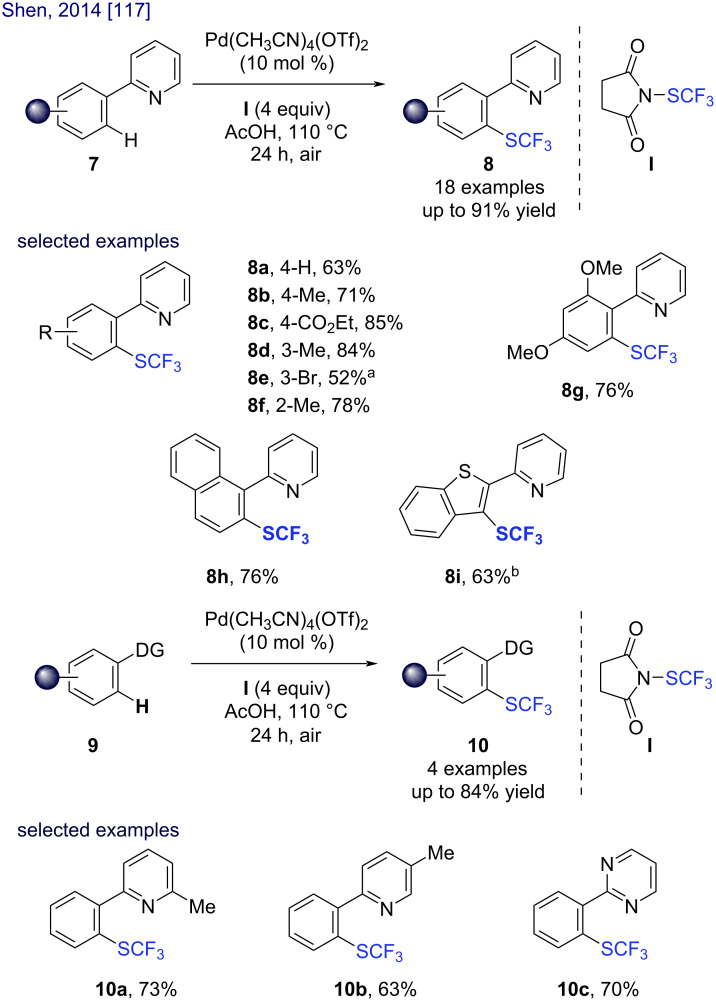
Trifluoromethylthiolation of 2-arylpyridine derivatives and analogs by means of palladium-catalyzed C–H activation reported by the group of Shen [117]. ^a^6 equiv of **I** were used at 150 °C for 24 h. ^b^The reaction was conducted at 150 °C for 24 h.

In 2014, Shen and Xu [117] developed a new methodology for the selective functionalization of 2-arylpyridine derivatives using an electrophilic SCF_3_ reagent, the Haas reagent **I** (Scheme 5) [118]. A broad range of 2-arylpyridine derivatives were trifluoromethylthiolated in good to high yields (18 examples, from 52 to 91% yields). The substitution pattern of the aromatic ring had no impact on the outcome of the reaction as illustrated with substrates substituted by a methyl group (**7b**, **7d**, and **7f**) at the *para*-, *meta*- and *ortho*-positions, which were readily functionalized in 71%, 84% and 78% yields, respectively. This reaction was also tolerant of a 2-naphthyl group, the palladium-catalyzed trifluromethylthiolation afforded the corresponding product **8h** in 76% yield. Also, the 2,4-dimethoxylated substrate **7g** and the benzothiophene derivative **7i** were successfully trifluoromethylthiolated in 76% and 63% yields, respectively. This reaction proved to be compatible with the presence of an ester (**8c**) or a halogen (**8e**). Other directing groups, such as substituted pyridines (**9a** and **9b**) and pyrimidine (**9c**) turned out to be also efficient in this transformation (Scheme 5, 4 examples, up to 84% yield).

**Scheme 6:**
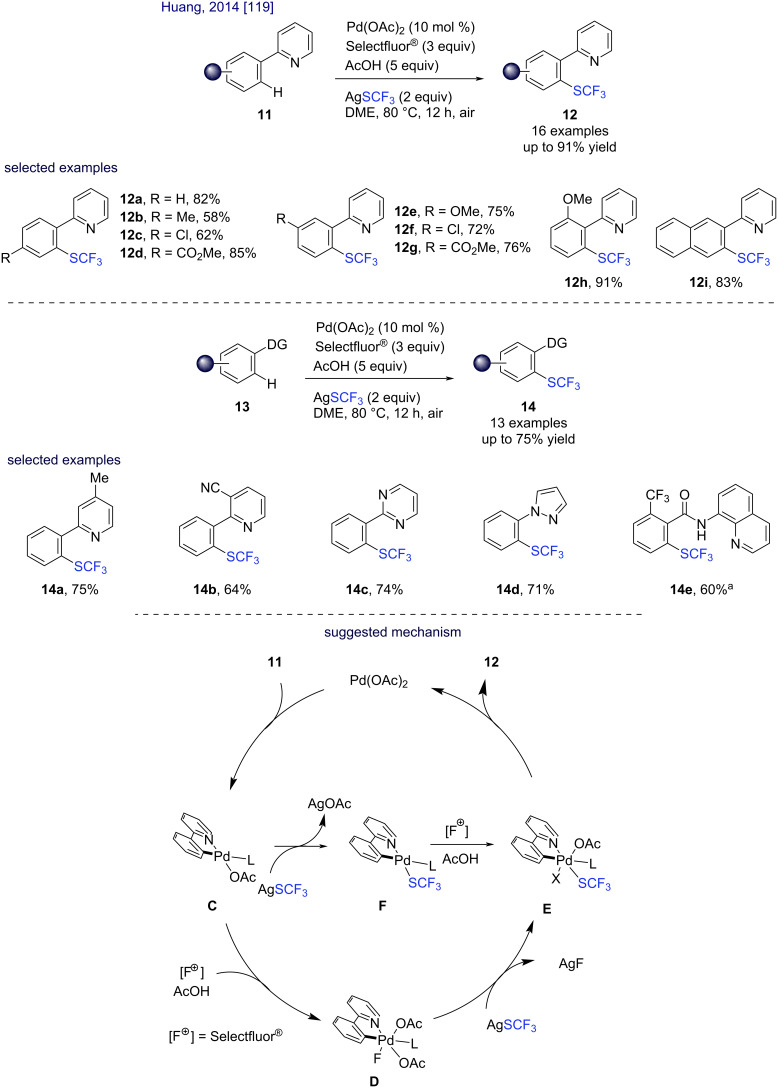
C(sp^2^)–SCF_3_ bond formation by Pd-catalyzed C–H bond activation using AgSCF_3_ and Selectfluor^®^ as reported by the group of Huang [119]. ^a^20 equiv of Cl_2_CHCOOH were used instead of AcOH.

The same year, the group of Huang reported an elegant and straightforward palladium(II)-catalyzed *ortho*-selective trifluoromethylthiolation of arenes bearing various directing groups using the nucleophilic trifluoromethylthiolating source AgSCF_3_ in combination with Selectfluor^®^ as oxidant (Scheme 6, 29 examples, up to 91% yield) [119]. 2-Arylpyridine derivatives bearing electron-donating groups, electron-withdrawing groups or halogen at the *para-* and *meta*-positions of the aromatic ring were readily functionalized (**11a–g**, 58–85% yields). Also 2-(2-methoxyphenyl)pyridine (**11h**) and 2-(2-naphthyl)pyridine (**11i**) were found to be suitable substrates leading to the corresponding products **12h** and **12i** in 91% and 83% yields, respectively. The use of other directing groups was also suitable for this transformation such as methyl and cyano-substituted pyridines **13a**,**b**, pyrimidine (**13c**), pyrazole (**13d**), as well as the amide derived from 8-aminoquinoline **13e** (Scheme 6, 13 examples, up to 75% yield).

In this study, two mechanisms were reported. The first one suggested that a palladacycle **C** is formed after the irreversible chelation of the 2-phenylpyridine substrate with palladium, which is the rate-determining step (KIE = 2.7). Subsequently palladacycle **C** is oxidized by Selectfluor^®^ to form a palladium(IV) complex **D**. After a ligand exchange with AgSCF_3_, the intermediate **E** is obtained, which, after reductive elimination, releases the desired product **12** and regenerates the catalyst. Alternatively, a ligand exchange with AgSCF_3_ occurs before the oxidation step, generating the palladium(II) complex **F**. After an oxidative addition in the presence of Selectfluor^®^, the palladium(IV) intermediate **E** is generated. Finally, after reductive elimination step, the desired product **12** is released and the catalyst regenerated. Note that, in this process, Selectfluor^®^ is playing a key role. Indeed, using this electrophilic fluorinating source as oxidant generates a Pd(IV)(ppy)F(OAc)_2_ (ppy = 2-phenylpyridine) complex as intermediate. As the competitive C–F bond formation was disfavored (slow reductive elimination step), the desired trifluoromethylthiolated product **12** is selectively afforded after a F/SCF_3_ ligand exchange.

**Scheme 7:**
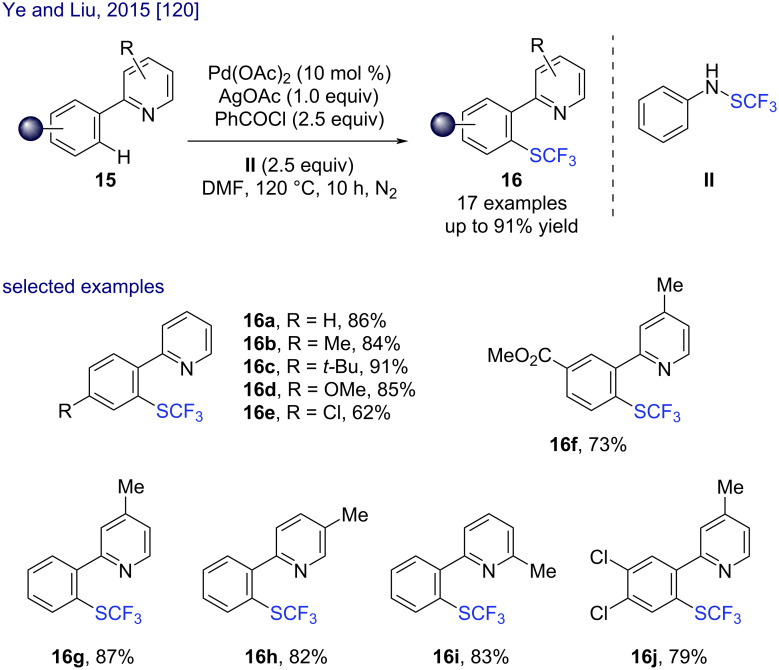
Palladium-catalyzed *ortho*-trifluoromethylthiolation of 2-arylpyridine derivatives reported by the group of Ye and Liu [120].

In 2015, Ye and Liu reported the palladium-catalyzed trifluoromethylthiolation of 2-arylpyridine derivatives using the Billard reagent **II** (Scheme 7) [120]. Unlike Shen's methodology (Scheme 5), the use of benzoyl chloride was necessary to activate the trifluoromethylthiolated reagent [120]. Unsubstituted and pyridines substituted derivatives **15** were very efficiently *ortho*-trifluoromethylthiolated (17 examples, up to 91% yield). This methodology was tolerant to electron-donating and electron-withdrawing groups as well as halogens (**16b**–**f**, 62–91% yields). The *meta*-substituted (**16f**) and disubstituted (**16j**) products were obtained in high yields (73% and 79%, respectively).

**Scheme 8:**
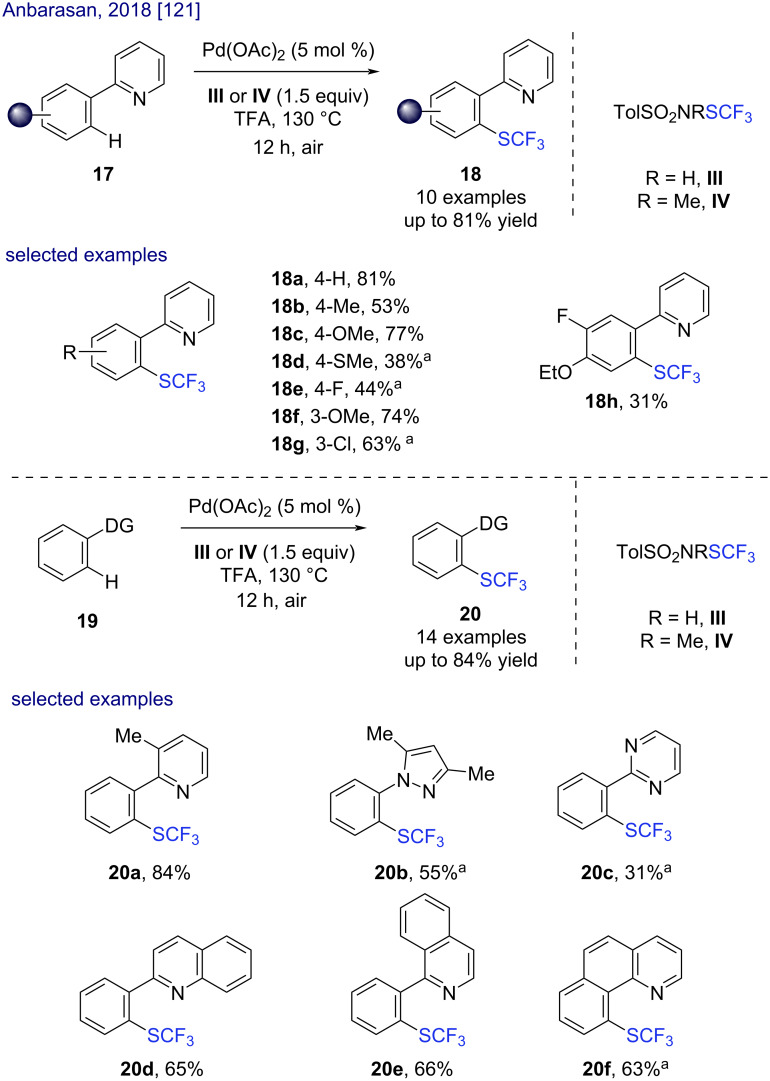
Palladium-catalyzed *ortho*-trifluoromethylthiolation of 2-arylpyridine and analogs reported by Anbarasan [121]. ^a^The reaction was conducted at 120 °C using 1.1 equiv of Billard reagent **IV** (R = Me).

In 2018, Anbarasan and co-workers described a palladium-catalyzed trifluoromethylthiolation of arenes by C–H bond activation bearing several directing groups (Scheme 8) [121]. With this methodology, the functionalization of 2-phenylpyridine derivatives was possible (10 examples, up to 81% yield) using the Billard reagents **III** or **IV** [122]. The trifluoromethylthiolation of 2-arylpyridines substituted by electron-donating groups such as methyl, methoxy or methylthio groups (**17b–d**) or by halogen (**17e**) was achieved (Scheme 8, up to 77% yield). Note that in case of disubstituted 2-(4-ethoxy-3-fluorophenyl)pyridine (**17h**), the expected product **18h** was isolated in 31% yield. Moreover, selective oxidation of the SCF_3_ residue into the corresponding sulfoxide and the sulfone was possible and the corresponding products were obtained in 98% and 95% yields, respectively.

This reaction was successfully expanded to the trifluoromethylthiolation of derivatives bearing various directing groups such as substituted pyridines (4 examples, up to 84% yield, **19a**, 84% yield), substituted pyrazole derivatives (6 examples, up to 55% yield, **19b**, 55% yield), pyrimidine (**19c**, 31% yield) as well as quinoline and isoquinoline (**19d** and **19e**, 65% and 66% yields, respectively). In addition, the trifluoromethylthiolated benzo[*h*]quinoline **20f** was obtained in good yield (63%).

**Scheme 9:**
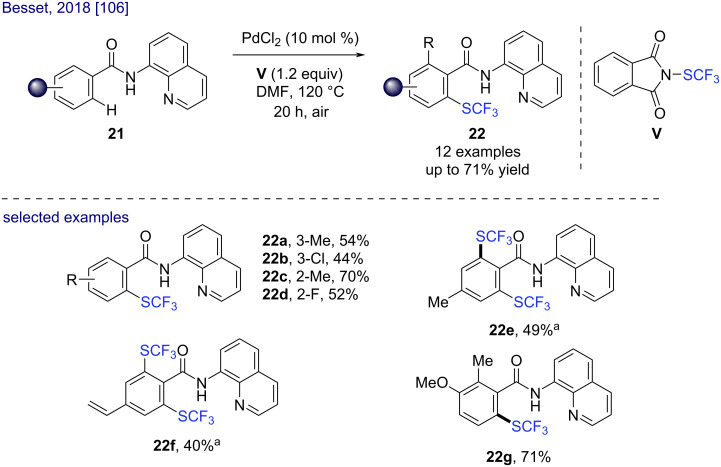
Mono- and ditrifluoromethylthiolation of benzamide derivatives derived from 8-aminoquinoline using PdCl_2_ as catalyst reported by Besset and co-workers [106]. ^a^2.2 equiv of the fluorinated source **V** were used.

The same year, Besset and co-workers reported a palladium-catalyzed C(sp^2^)–SCF_3_ bond formation on amides derived from 8-aminoquinoline as a cleavable directing group in the presence of the Munavalli reagent **V** (Scheme 9, 12 examples, up to 71% yield) [106]. Depending on the substitution pattern on the aromatic ring, the amides were mono- or difunctionalized. Indeed, *meta*- and *ortho*-substituted derivatives (**21a**–**d**) were selectively trifluoromethylthiolated while *para*-substituted substrates led to the difunctionalized products **22e** and **22f**. Within these reaction conditions, the polysubstituted derivative **21g** was also functionalized in high yield (71%).

Pleasingly, other metals have been also successfully applied for the trifluoromethylthiolation of aromatic derivatives by C(sp^2^)–H bond activation such as Rh(III) and Co(III)-based catalysts as depicted below.

**Scheme 10:**
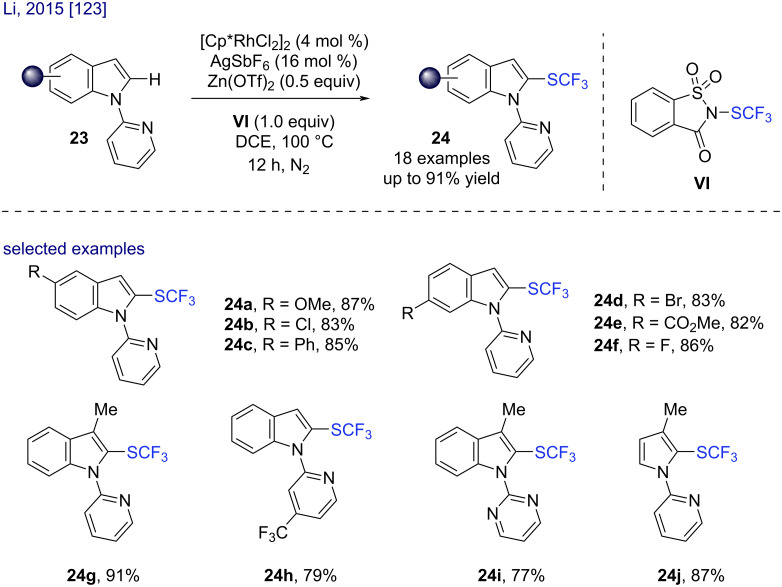
Regioselective Cp*Rh(III)-catalyzed directed trifluoromethylthiolation reported by the group of Li [123]. Cp* = pentamethylcyclopentadienyl.

**Rhodium catalysis:** In 2015, the group of Li disclosed the Cp*Rh(III)-catalyzed regioselective trifluoromethylthiolation of *N*-substituted indoles with (substituted) pyridines or pyrimidine as the directing groups (Scheme 10) [123]. The selective trifluoromethylthiolation of indoles at the C2 position was achieved in the presence of *N*-(trifluoromethylthio)saccharine (**VI**, Shen’s reagent) as both oxidant and electrophilic source (18 examples, up to 91% yield). Indoles bearing various electron-donating and electron-withdrawing groups as well as halogens at the C5-position and at the C6-position were functionalized in high yields (**24a**–**f**, 82–87% yields). The substitution of the indole at the C3-position did not impact the reaction and the product **24g** was obtained in 91% yield. Substituted pyridines and pyrimidine (**24h** and **24i**) were also used as directing groups (7 examples, up to 86% yield). This methodology was extended to the functionalization of other heteroaromatic derivatives (**24j**, 87% yield). It should be noted that the presence of zinc triflate, a Lewis acid, was used for the activation of the electrophilic source **VI**.

**Scheme 11:**
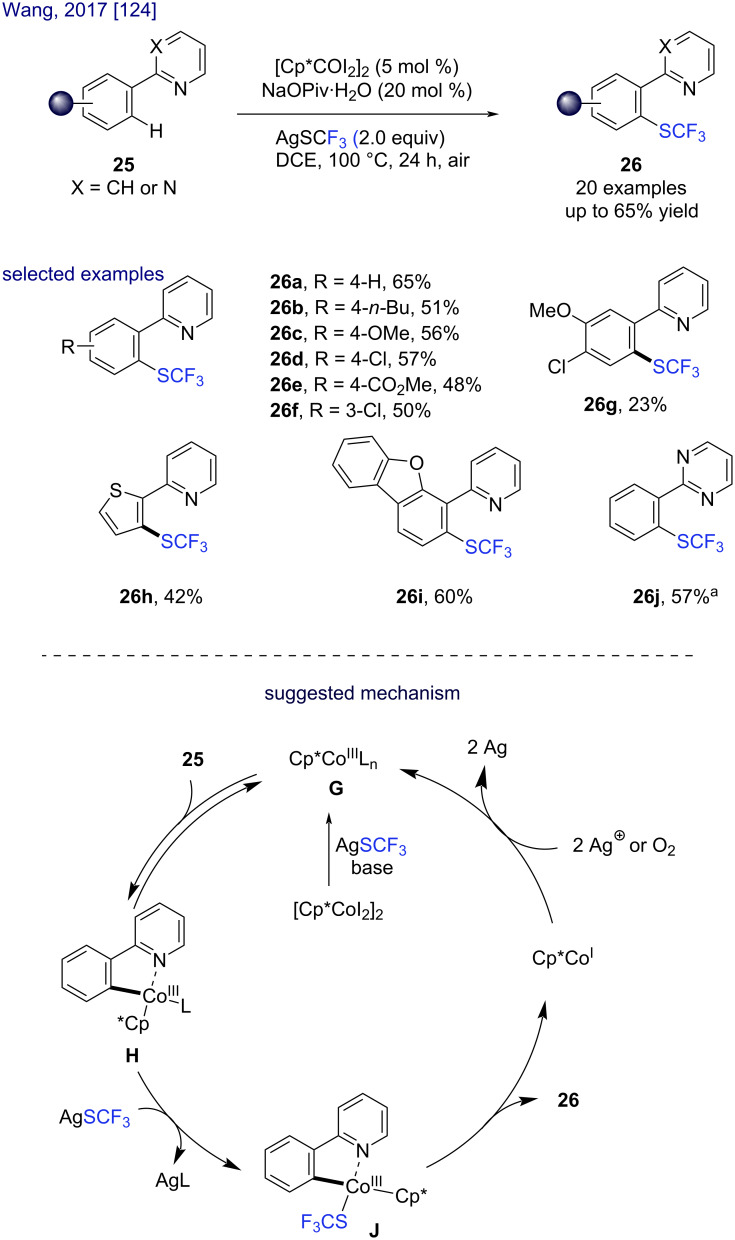
Cp*Co(III)-catalyzed *ortho*-trifluoromethylthiolation of 2-phenylpyridine and 2-phenylpyrimidine derivatives reported by Wang and co-workers [124]. ^a^CH_2_Cl_2_ was used as solvent.

**Cobalt catalysis:** In 2017, Wang described the Cp*Co(III)-catalyzed trifluoromethylthiolation of 2-phenylpyridine derivatives using AgSCF_3_ (Scheme 11) [124]. This methodology allowed the functionalization of several aromatic compounds bearing a pyridine or a pyrimidine as a directing group (20 examples, up to 65% yield). The reaction proceeded smoothly with substrates bearing an electron-donating group (**25b**,**c**), halogen (**25d**) or withdrawing group (**25e**) and the desired SCF_3_-containing products were obtained in moderate to good yields. The functionalization of trisubstituted arene **25g** and heteroarene **25h** was also possible leading to the corresponding products **26g** and **26h** in moderate yields (23% and 42% yields, respectively). Regarding the reaction mechanism, the active Co(III) complex **G** was obtained from the dimeric catalyst [Cp*CoI_2_]_2_ in the presence AgSCF_3_ and/or NaOPiv·H_2_O. Then, the reversible formation of the metallacycle **H** occurs, which after a ligand exchange in the presence of AgSCF_3_ leads to the formation of adduct **J**. The product **26** is released via a reductive elimination step, generating at the same time the reduced cobalt Cp*Co(I), which is converted to the active catalyst after oxidation.

**Scheme 12:**
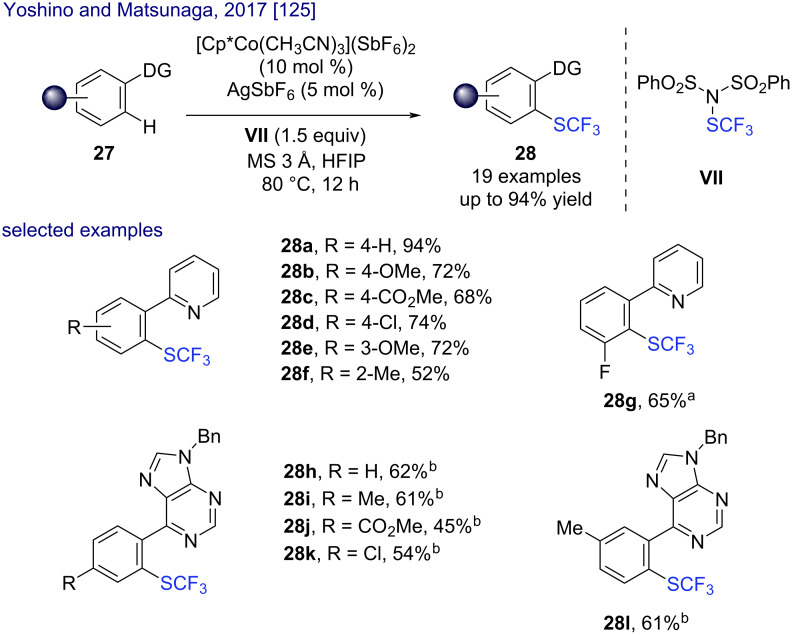
Cp*Co(III)-catalyzed *ortho*-trifluoromethylthiolation of 2-phenylpyridine and 6-phenylpurine derivatives described by Yoshino and Matsunaga [125]. ^a^Without AgSbF_6_. ^b^2.0 equiv of **VII**, 10 mol % of AgOAc and 30 mol % of Gd(OTf)_3_ were used instead of AgSbF_6_.

The same year, Yoshino and Matsunaga described a similar methodology, using the cobalt(III) complex [Cp*Co(CH_3_CN)_3_](SbF_6_)_2_ and *N*-trifluoromethylthiodibenzenesulfonimide **VII** as electrophilic SCF_3_ source (Scheme 12) [125]. Under these reaction conditions, 2-arylpyridines (9 examples, up to 94% yield) or 6-arylpurines (10 examples, up to 72% yield) were *ortho*-trifluoromethylthiolated. The substitution pattern of the aryl part did not impact the outcome of the reaction. This methodology was also tolerant to a large range of functional groups (ester, halogen) as illustrated by the products **28c**, **28j**, **28d**, **28k**, and **28g**.

#### I.2) Transition-metal-catalyzed C–H trifluoromethylthiolation of vinylic C(sp^2^) centers

Several research groups have been interested in the development of strategies for the formation of vinylic C(sp^2^)–SCF_3_ bonds, offering an efficient tool towards the synthesis of challenging *Z*-isomers.

**Scheme 13:**
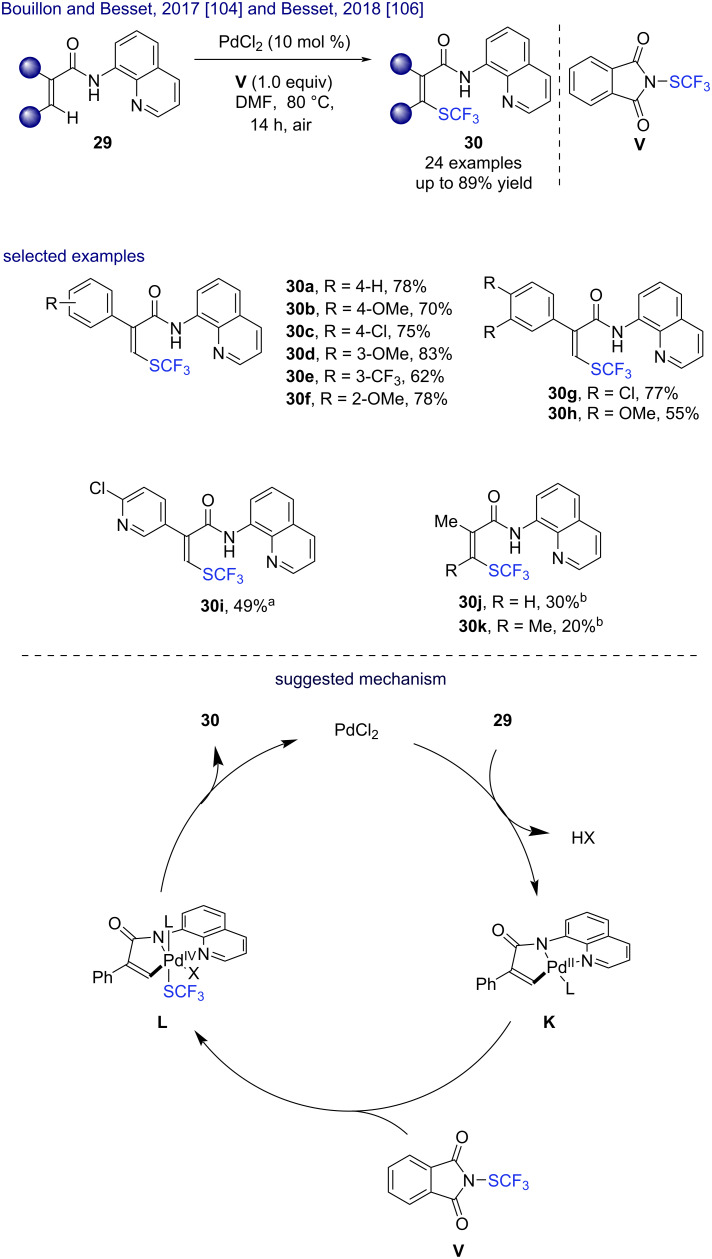
Diastereoselective trifluoromethylthiolation of acrylamide derivatives derived from 8-aminoquinoline using PdCl_2_ reported by Bouillon and Besset [104,106]. ^a^20 mol % of PdCl_2_ and 2.0 equiv of SCF_3_ source **V** were used for 36 h. ^b^The reaction was conducted with 30 mol % of PdCl_2_ and 2.0 equiv of reagent **V** were used.

**Palladium catalysis:** In 2018, Bouillon and Besset described the first example of a selective palladium-catalyzed trifluoromethylthiolation of α-arylacrylamides derived from 8-aminoquinolines **29** by C–H bond activation (Scheme 13) [104]. Using the Munavalli reagent **V** as an electrophilic SCF_3_ source, this diastereoselective method selectively led to the formation of *Z*-isomers and turned out to be robust (not air or moisture sensitive). Under these mild reaction conditions, a panel of α-(hetero)arylacrylamides were trifluoromethylthiolated in good to high yields. Acrylamides substituted at the α-position by an aryl bearing an electron-donating group (OMe) or halogen (Cl) at the *para*-position were readily functionalized (**30b** and **30c**, 70% and 75% yields, respectively). The substitution of the arene with a CF_3_ residue at the *meta*-position or with a methoxy group at the *ortho*-position did not have any impact to the outcome of the reaction (**30e** and **30f**, 62% and 78% yields, respectively). It should be noted that acrylamides bearing a disubstituted arene (**29g**,**h**) and an heteroaryl (**29i**) at the α-position were also suitable substrates for this reaction. Finally, acrylamides bearing a methyl group at the α-position (**29j**) or the α,β-dimethylated acrylamide (**29k**) were suitable substrates albeit the corresponding products were obtained in 30% and 20% yields, respectively. Several mechanistic experiments revealed that the C(sp^2^)–H bond activation step was reversible and represented the rate-determining step (KIE = 2.4). First, the chelation of the palladium(II) catalyst with the bidentate directing group, followed by the C(sp^2^)–H bond activation involving a concerted metalation–deprotonation pathway affords the metallacycle **K**. After an oxidative addition in the *N*-SCF_3_ bond of the Munavalli reagent **V**, the palladium(IV) species **L** is obtained. Finally, the reductive elimination affords the product and regenerates the catalyst. The same year, Besset and co-workers extended this methodology to a larger class of acrylamides [106].

#### I.3) Transition-metal-catalyzed trifluoromethylthiolation of aliphatic C(sp^3^)–H bonds

Despite the important progresses presented in the previous section, some limitations of the trifluoromethylthiolation persist. In particular, the functionalization of a C(sp^3^)–H bond with a trifluoromethylthiolated moiety by transition-metal-catalyzed C–H activation remains a challenging task both in terms of reactivity and selectivity.

**Scheme 14:**
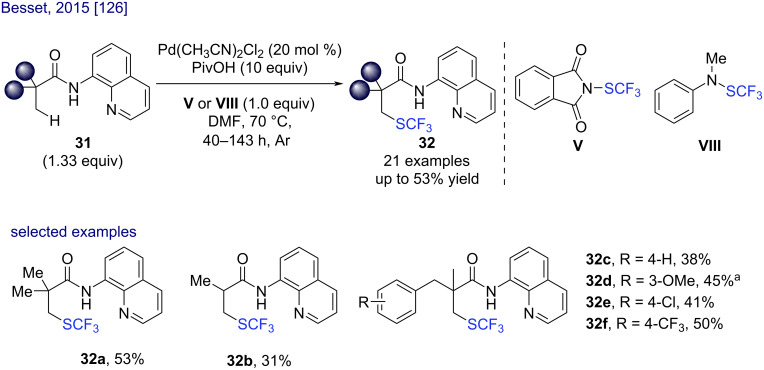
C(sp^3^)–SCF_3_ bond formation on aliphatic amide derivatives derived from 8-aminoquinoline by palladium-catalyzed C–H bond activation described by Besset and co-workers [126]. Product **32d** was contaminated with 10% of an inseparable impurity.

In 2015, Besset reported the first C(sp^3^)–SCF_3_ bond formation of unactivated primary C(sp^3^) centers by transition-metal-catalyzed C–H activation with the Munavalli or the Billard reagents as the trifluoromethylthiolation source (Scheme 14) [126]. Using a bidentate directing group, this methodology allowed the functionalization of a large range of aliphatic amides with a primary β-C(sp^3^)–H bond (21 examples, up to 53% yield). The methodology was applied to the functionalization of a series of amides having an α-quaternary center (α,α-dialkyl (**31a**), α-alkyl,α-benzyl derivatives **31c**–**f**) as well as to an amide with an α-tertiary center (**31b**) and pleasingly, the presence of α-C–H bonds did not have a significant impact on the outcome of the reaction. It should be noted that this methodology afforded the products with a high regioselectivity, and no incorporation of the SCF_3_ moiety on the benzylic or at the C5 position of the quinoline part of the directing group was observed. Note that in 2018, Besset and Lebel developed a more efficient process for the palladium-catalyzed trifluoromethylthiolation by C**–**H bond activation under continuous flow conditions [127].

#### I.4) Difluoromethylthiolation of aromatic and vinylic C(sp^2^)–H bonds (C–SCF_2_H and C–SCF_2_CO_2_Et bonds)

More recently, researchers became interested in the synthesis of molecules substituted with original and functionalized fluorinated moieties such as SCF_2_FG [128–136] (FG = functional group). In sharp contrast with the trifluoromethylthiolation reaction, only a handful of reports dealt with the incorporation of such high value-added fluorinated moieties onto C(sp^2^) centers by transition-metal-catalyzed C–H bond activation.

**Scheme 15:**
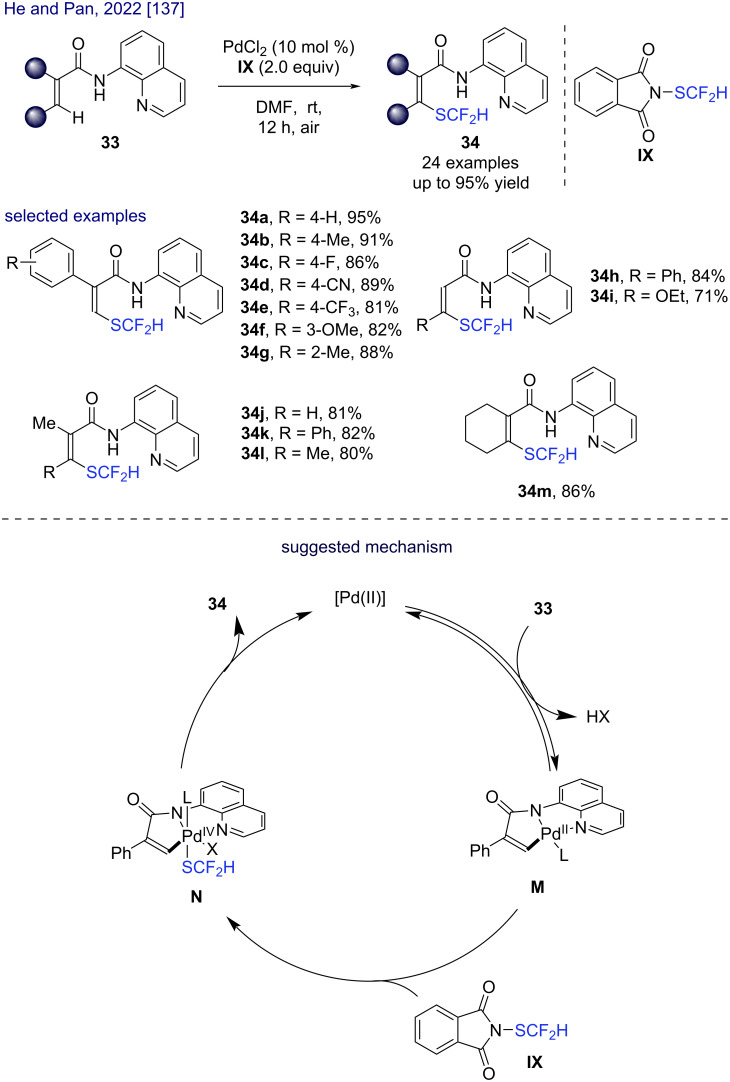
Regio- and diastereoselective difluoromethylthiolation of acrylamides under palladium catalysis reported by He and Pan [137].

In 2022, He and Pan reported the first example of a difluoromethylthiolation achieved by transition-metal-catalyzed C–H bond activation (Scheme 15) [137]. Using the reagent **IX** and a catalytic amount of PdCl_2_, they succeeded in the functionalization of acrylamides **33** derived from 8-aminoquinoline. Within these mild conditions, α-arylacrylamides substituted at *para-*, *meta-*, and *ortho*-positions were readily difluoromethylthiolated (**34a**–**g**, 81–95% yields). This reaction was also tolerant to a large class of functional groups (halogens, cyano, trifluoromethyl), affording products **34c**,**d** and **34e** in high yields (86%, 89% and 81%, respectively). The α-methyl-substituted acrylamide **33j** also underwent difluoromethylthiolation to give product **34j** in 81% isolated yield. The α,β- and β-substituted acrylamides were functionalized in high yields (**34h**,**i** and **34k–m**, 71–86%). The plausible mechanism is similar as the one reported by Besset for the trifluoromethylthiolation of acrylamides derived from 8-aminoquinoline (Scheme 13).

**Scheme 16:**
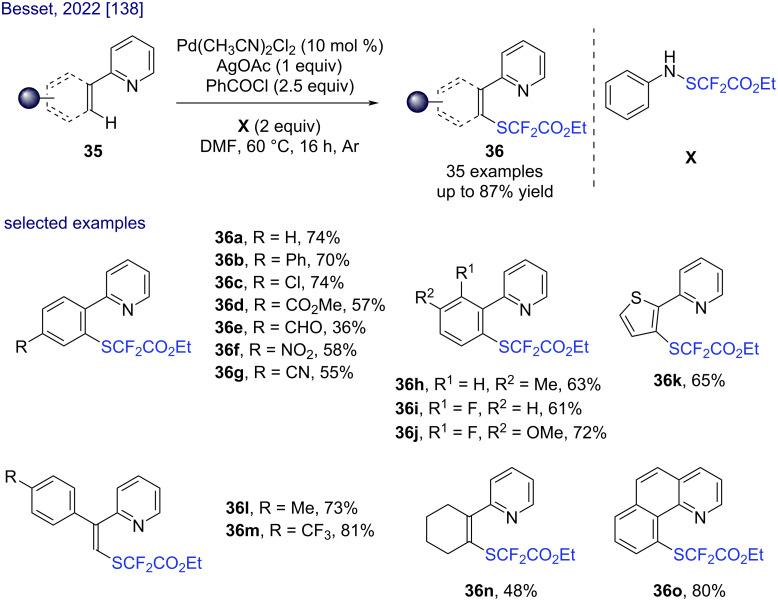
Palladium-catalyzed (ethoxycarbonyl)difluoromethylthiolation reaction of 2-(hetero)aryl and 2-(α-aryl-vinyl)pyridine derivatives reported by Besset [138].

The same year, Besset and co-workers reported the first palladium-catalyzed C(sp^2^)–SCF_2_CO_2_Et bond formation by C–H bond activation (Scheme 16) [138]. In the presence of the electrophilic SCF_2_CO_2_Et source **X**, the methodology was successfully applied for the functionalization of 2-arylpyridine derivatives **35a**–**i** as well as 2-vinylpyridine derivatives **35j**–**m** (35 examples, up to 87% yield). The substitution pattern of the aryl substituent of the 2-phenylpyridine derivatives did not influence the reaction as for instance products **36b**, **36h**, and **36i** were obtained in good yields (70%, 63% and 61% yield, respectively). This reaction was tolerant to a broad range of functional groups such as halogens, ester, aldehyde, cyano, and nitro (**36c**–**g**, 36–74% yield). It is noteworthy that a disubstituted compound **35j** and a thiophene derivative **35k** were also efficiently difluoromethylthiolated (**36j** and **36k**, 72% and 65%, respectively). α-Substituted vinylpyridines with electron-donating or electron-withdrawing groups on the aromatic ring were functionalized and **36l** and **36m** were easily isolated in 73% and 81% yields, respectively. Even an α,β-disubstituted vinylpyridine **35n** and the benzoquinoline **35o** were smoothly functionalized showing the efficiency of the approach. Of high interest, the modularity of the SCF_2_CO_2_Et was highlighted by its conversion into various other fluorinated residues (amide, carboxylic acid) and its selective oxidation into the corresponding sulfoxide and sulfone.

#### I.5) Trifluoromethylselenolation of aromatic and vinylic C(sp^2^)–H bonds by palladium catalysis

**Scheme 17:**
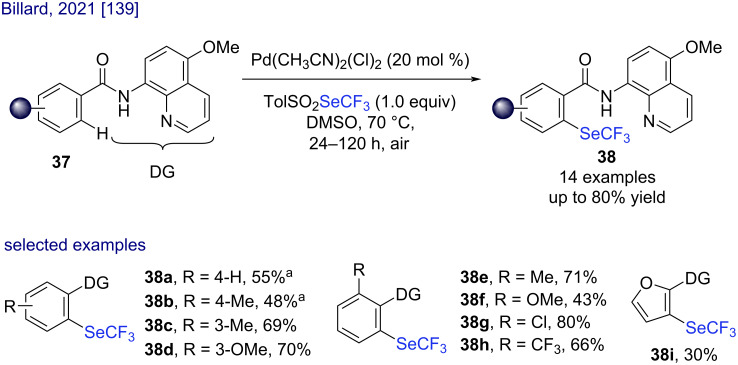
Pd(II)-catalyzed trifluoromethylselenolation of benzamides derived from 5-methoxy-8-aminoquinoline reported by the group of Billard [139]. ^a^The yields given are the sum of the yields of mono- and ditrifluoromethylselenolated products.

Very recently, the palladium-catalyzed trifluoromethylselenolation of (hetero)aromatic and olefinic derivatives has been investigated by the group of Billard using similar catalytic systems as those depicted for the trifluoromethylthiolation reactions. Indeed, using amides **37** derived from 5-methoxy-8-aminoquinoline, the functionalization of (hetero)aromatic compounds was achieved using 20 mol % of Pd(CH_3_CN)_4_Cl_2_ in the presence of TolSO_2_SeCF_3_ as the fluorinating source (14 examples, Scheme 17) [139]. Of note, when the reaction was carried out on derivatives bearing substituents at the *meta-* (**37c** and **37d**) as well as at the *para-* (**37a** and **37b**) positions, the corresponding products **38a–d** were obtained as a mixture of mono- and disubstituted trifluoromethylselenolated derivatives with global yields ranging from 48% to 70%. When one of the *ortho*-positions of the aromatic ring was substituted with a Me (**37e**), a OMe (**37f**), a Cl (**37g**) or a CF_3_ (**37h**) group, the corresponding compounds **38e**, **38f**, **38g**, and **38h** were isolated in moderate to high yields (43% to 80%). The methodology also allowed the trifluoromethylselenolation of the furan derivative **37i**, which led to the desired product **38i** in 30% yield. A careful monitoring of the reaction unveiled the rapid formation of the CF_3_SeSeCF_3_ dimer, which could be the active trifluoromethylselenolating reagent in this transformation.

**Scheme 18:**
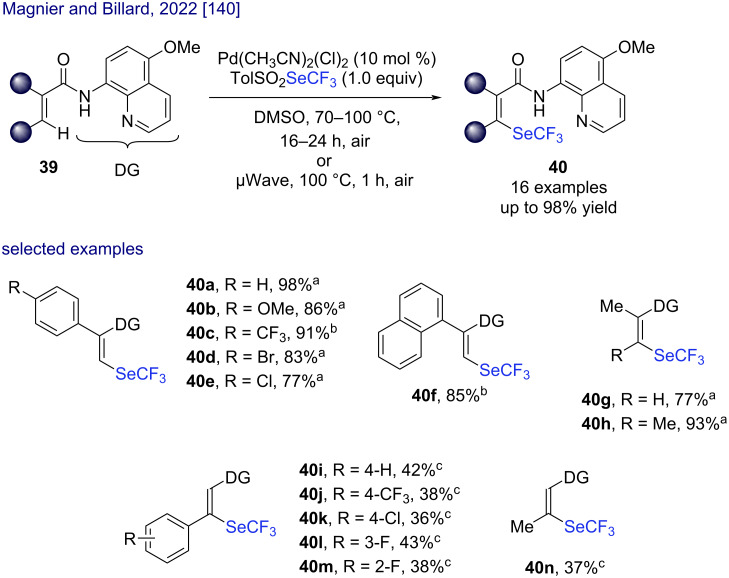
Pd(II)-catalyzed trifluoromethylselenolation of acrylamide derivatives derived from 5-methoxy-8-aminoquinoline reported by the groups of Magnier and Billard [140]. ^a^Microwave, 100 °C, 1 h. ^b^70 °C for 16 h. ^c^20 mol % of Pd(CH_3_CN)_2_(Cl)_2_, 100 °C for 24 h.

In 2022, Magnier, Billard and co-workers applied the previous methodology to the trifluoromethylselenolation of acrylamide derivatives [140]. Using the same directing group, a panel of α-arylacrylamide derivatives **39a–f** was successfully functionalized with a high *Z*-selectivity (yields up to 98%, Scheme 18). Both, thermal reaction conditions (DMSO at 70 °C for 16 h) and microwave irradiation (100 °C using microwaves in only 1 h) turned out to be efficient in the process. α-Methyl- and α,β-dimethylacrylamides **39g** and **39h** were also functionalized. Furthermore, a series of β-substituted acrylamides **39i–m** with various substituents readily underwent the trifluoromethylselenolation reaction with high selectivity in moderate yields. Finally, the SeCF_3_-containing β-methylacrylamide **40n** was also obtained in 37% yield.

### II. Transition-metal-catalyzed fluoroalkoxylation of (hetero)arenes by C–H bond activation

#### II.1) Fluoroalkoxylation of aromatic C(sp^2^)–H bonds by transition-metal catalysis

Over the last years, key advances have been made for the formation of a C(sp^2^)–OCHRCF_3_ bond by transition-metal-catalyzed C–H bond activation. Indeed, fluorinated ethers [71,141–153] are key compounds, with especially molecules substituted with the 2,2,2-trifluoroethoxy moiety (OCH_2_CF_3_), an important fluorinated group found in several bioactive compounds such as flecanide [154–155] and lansoprazole [156], as flagship molecules. Although the transition-metal-catalyzed hydroxylation and alkoxylation have been studied especially under palladium catalysis [157–158], the direct dehydrogenative 2,2,2-trifluoroethoxylation of (hetero)arenes, often using 2,2,2-trifluoroethanol as a readily available, inexpensive, and green fluorination source [159–160], is still underexplored.

**Scheme 19:**
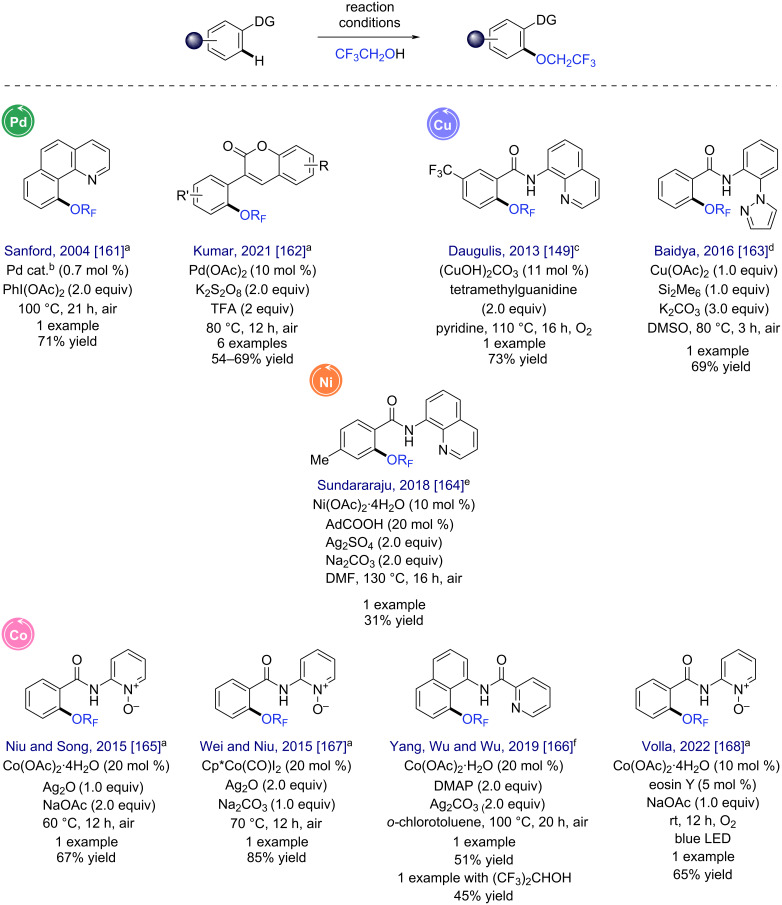
Transition-metal-catalyzed dehydrogenative 2,2,2-trifluoroethoxylation of (hetero)aromatic derivatives by C–H bond activation [149]. ^a^CF_3_CH_2_OH was used as solvent. ^b^Pd cat. = [Pd(μ-κ^1^-OAc)(κ^2^-N,C_10_-benzo[*h*]quinoline)]_2_. ^c^5 equiv of CF_3_CH_2_OH. ^d^15 equiv of CF_3_CH_2_OH. ^e^50 equiv of CF_3_CH_2_OH. ^f^CF_3_CH_2_OH or (CF_3_)_2_CHOH was used as solvent with *o*-chlorotoluene with a ratio of 1:1. AdCOOH = adamantanecarboxylic acid, DMAP = 4-(dimethylamino)pyridine, OR_F_ = OCH_2_CF_3_.

In 2004, Sanford and co-workers reported the dehydrogenative 2,2,2-trifluoroethoxylation of benzo[*h*]quinoline under palladium catalysis in the presence of PhI(OAc)_2_ as oxidant (Scheme 19) [161]. Since this seminal work and in the course of their investigation towards the development of new methods for the alkoxylation of C(sp^2^) centers by transition-metal catalysis, few examples of transition-metal-catalyzed dehydrogenative 2,2,2-trifluoroethoxylation reactions have been reported. In 2021, the palladium-catalyzed *ortho*-2,2,2-trifluoroethoxylation of 3-arylcoumarins was depicted by the group of Kumar (6 examples, up to 69% yield) [162]. Further developments unveiled the use of copper catalysts for such functionalization. In 2013, the group of Daugulis described the copper-catalyzed *ortho*-2,2,2-trifluoroethoxylation of a 3-trifluoromethylated benzamide derived from 8-aminoquinoline, giving the corresponding product in 73% yield [149]. The group of Baidya showed that the dehydrogenative 2,2,2-trifluoroethoxylation of benzamide with another bidentate directing group was also possible in the presence of Cu(OAc)_2_ and hexamethyldisilane [163]. Using *N,N*- and *N,O*-bidentate directing groups, the construction of C(sp^2^)–OCH_2_CF_3_ bonds by C**–**H bond activation was also reported using Ni [164] and Co catalysis [165–167]. In 2022, Volla and co-workers reported the *ortho*-2,2,2-trifluoroethoxylation of benzamide using an *N,O*-bidentate directing group by merging Co- and visible light organophotocatalysis [168].

**Scheme 20:**
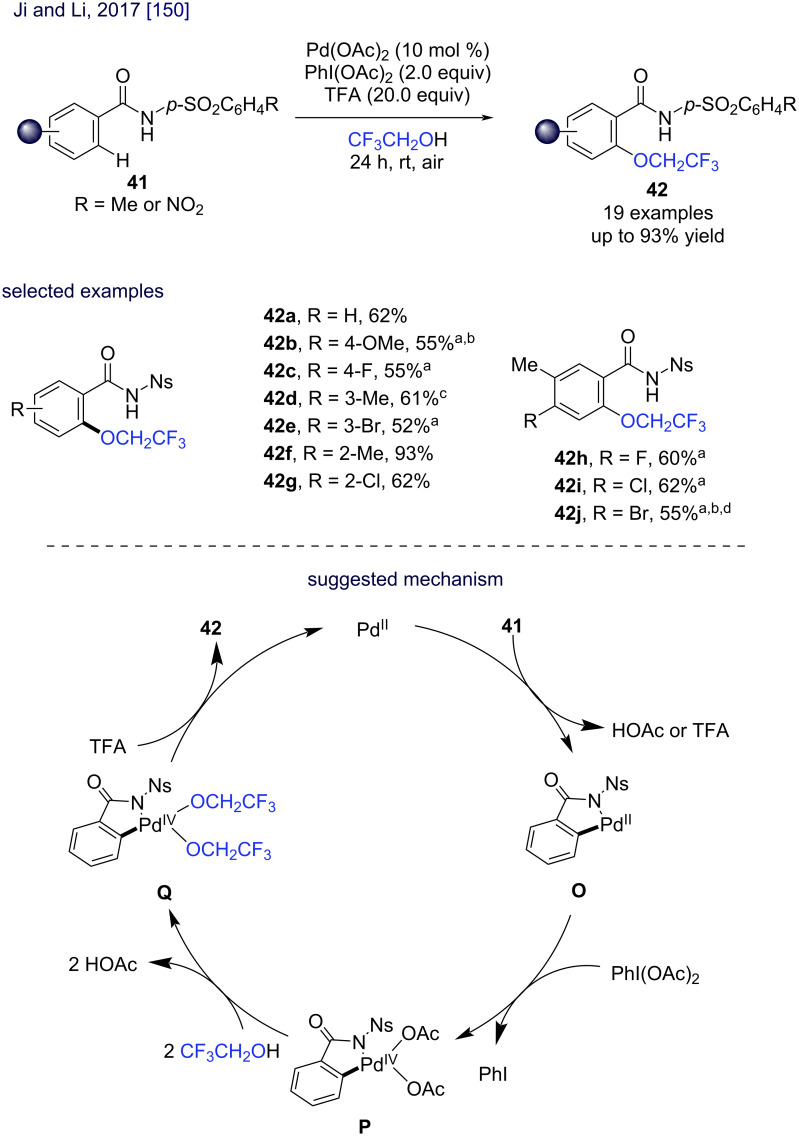
Pd(II)-catalyzed *ortho*-2,2,2-trifluoroethoxylation of *N*-sulfonylbenzamides reported by the group of Ji and Li [150]. ^a^50 °C. ^b^15 equiv TFA. ^c^5 equiv TFA. ^d^*N*-acetylglycine (60 mol %). Ns: 4-nitrobenzenesulfonyl.

**Palladium catalysis:** In 2017, Ji, Li and co-workers reported a thorough study on the Pd-catalyzed 2,2,2-trifluoroethoxylation of *N*-sulfonylbenzamides in the presence of PhI(OAc)_2_ as oxidant and an excess of TFA (Scheme 20) [150]. The functionalization of diversely substituted derivatives bearing either electron-donating groups (**41b**, **41d**, and **41f**) and electron-withdrawing groups (**41c**, **41e**, and **41g**) was achieved (19 examples, up to 93% yield). Of note, the transformation was also efficient with disubstituted substrates such as **41h**–**j**. The authors suggested the following mechanism. After formation of the metallacycle **O**, the latter is oxidized leading to the Pd(IV) species **P**. After a ligand exchange, the intermediate **Q** is generated. Finally, a reductive elimination step affords the expected functionalized product **42** and regenerates the catalyst.

**Scheme 21:**
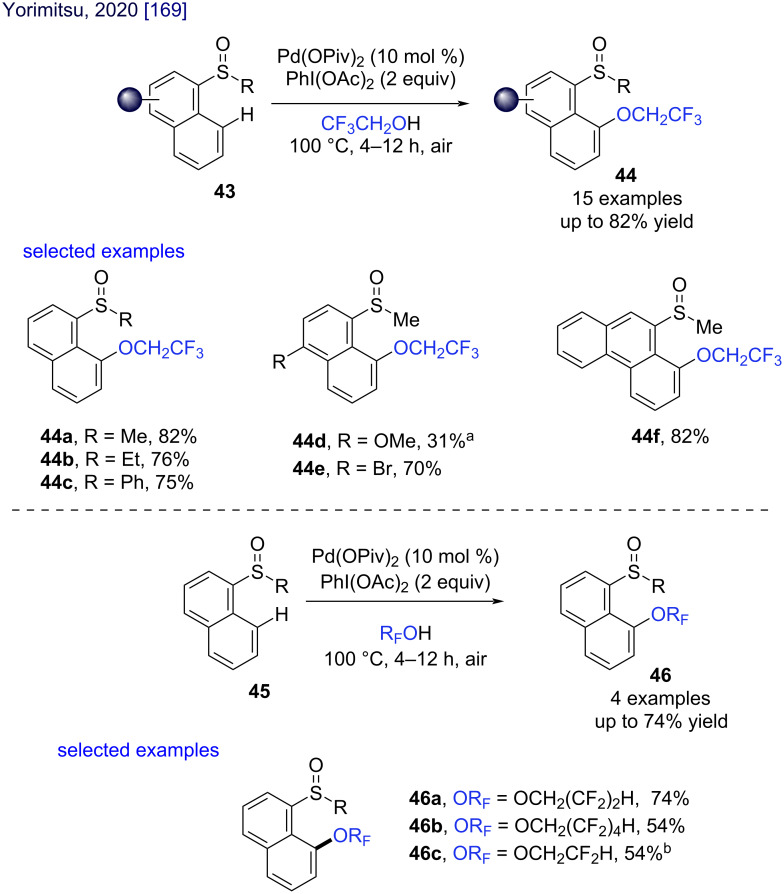
Pd(II)-catalyzed selective 2,2,2-trifluoroethoxylation and other fluoroalkoxylations of naphthalene sulfoxide derivatives reported by the group of Yorimitsu [169]. ^a^80 °C for 1 h. ^b^R_F_OH/AcOH 1:7.

The 2,2,2-trifluoroethoxylation reaction is not restricted to amides. In 2020, Yorimitsu and co-workers developed a methodology allowing the formation of a C(sp^2^)–OR_F_ bond through palladium-catalyzed C–H bond activation [169]. Using Pd(OPiv)_2_ as the catalyst in the presence of PhI(OAc)_2_ (PIDA), the naphthalene sulfoxide **43a** was 2,2,2-trifluoroethoxylated in 82% yield (Scheme 21). The substituent of the sulfoxide part does not impact the efficiency of the reaction as illustrated by the synthesis of compounds **44b** and **44c**. The presence of an electron-donating substituent in the *para*-position of the directing group was found to be deleterious for the reaction since **44d** was obtained in 31% yield while its brominated analog **44e** was isolated in 70% yield. Mechanistic studies indicated that the C–H bond activation event was the rate-limiting step and the authors suggested a similar mechanism to the one depicted in Scheme 20: formation of a palladacycle thanks to a concerted metalation deprotonation (CMD) process followed by oxidation, ligand exchange with CF_3_CH_2_OH, and finally, reductive elimination affording the expected product and regenerating the catalyst. Gratifyingly, the approach was applied to the incorporation of other fluorinated moieties such as OCH_2_CF_2_H and OCH_2_(CF_2_)*_n_*H (*n* = 2 or 4) and gave compounds **46a–c**.

Thanks to the transient directing group strategy [35,170–182], efficient methodologies for the functionalization of previously reluctant compounds such as benzaldehyde derivatives were developed, and key advances are depicted below.

**Scheme 22:**
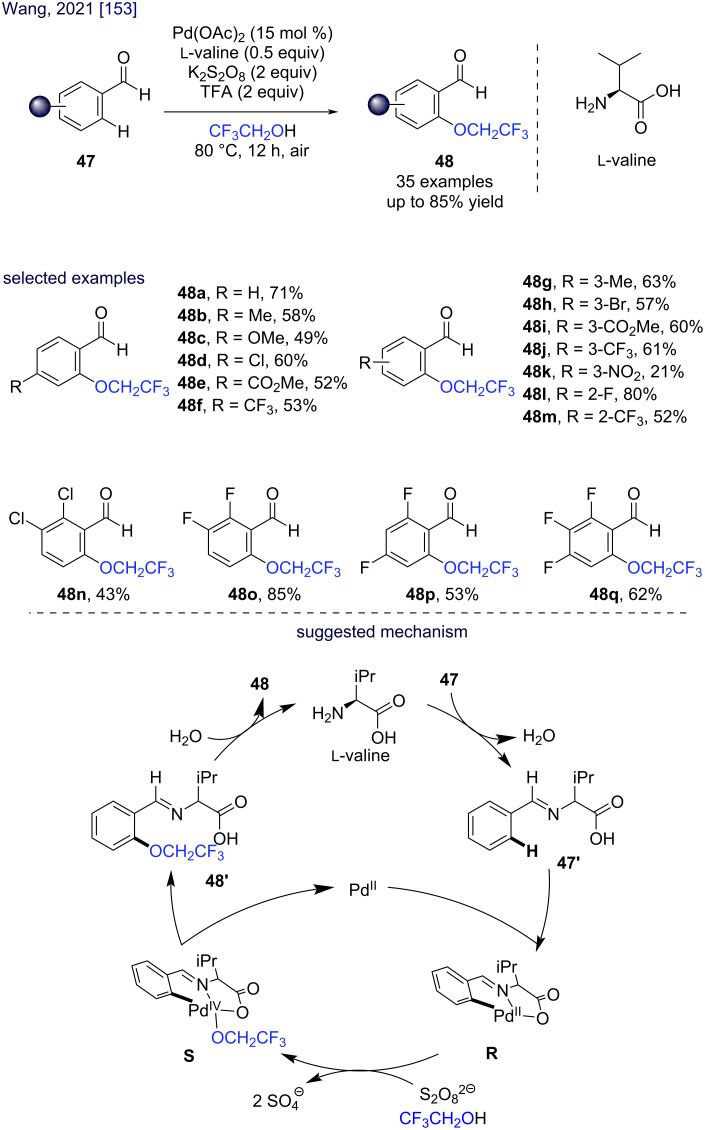
Pd(II)-catalyzed selective *ortho*-2,2,2-trifluoroethoxylation of benzaldehyde derivatives by means of the transient directing group strategy as reported by the group of Wang [153].

In 2021, Wang and co-workers described the selective palladium-catalyzed *ortho*-2,2,2-trifluoroethoxylation of a series of benzaldehydes (Scheme 22, 35 examples) using the amino acid ʟ-valine in the presence of K_2_S_2_O_8_ and TFA at 80 °C [153]. This reaction proved to be highly tolerant to various substituents including a CF_3_ group at the *ortho-, meta-* and *para*-positions (**48m**, **48j**, and **48f**, respectively), halogens (**48d**, **48h**, and **48l**), an ester moiety (**48e** and **48i**), and a methoxy group (**48c**). Note that even di- and trisubstituted benzaldehydes **47n**–**q** were smoothly functionalized under these conditions. The authors also suggested a plausible mechanism. The amino acid acts as an organocatalyst and first reacts with the benzaldehyde **47** to generate the transient directing group (**47’**). Then, formation of the palladacycle (species **R**) followed by its oxidation to a Pd(IV) intermediate and a ligand exchange with 2,2,2-trifluoroethanol leads to the formation of the species **S**. The latter complex **S** undergoes a reductive elimination leading to the compound **48’** along with the regeneration of the palladium catalyst. Finally, after hydrolysis of **48’**, the expected product **48** is afforded together with the organocatalyst.

**Scheme 23:**
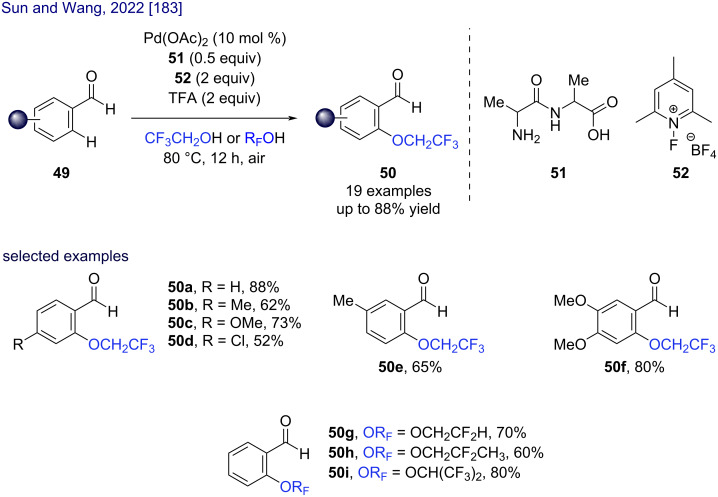
Pd(II)-catalyzed selective *ortho*-2,2,2-trifluoroethoxylation (and other fluoroalkoxylations) of benzaldehyde derivatives via the assistance of a transient directing group reported by the group of Sun and Wang [183].

Then, the group of Sun and Wang used a similar approach for the 2,2,2-trifluoroethoxylation of benzaldehydes under palladium catalysis using the amino acid **51** as organic catalyst in the presence of the fluoropyridinium salt **52** (19 examples, up to 88% yield, Scheme 23) [183]. Pleasingly, the methodology was extended to the formation of C(sp^2^)–OR_F_ bonds starting from benzaldehyde (OR_F_ = 2,2-difluoroethoxy **50g**, 2,2-difluoropropoxy **50h**, and 1,1,1,3,3,3-hexafluoroisopropoxy **50i**).

#### II.2) Fluoroalkoxylation of aliphatic C(sp^3^)–H bonds by transition-metal-catalysis

**Scheme 24:**
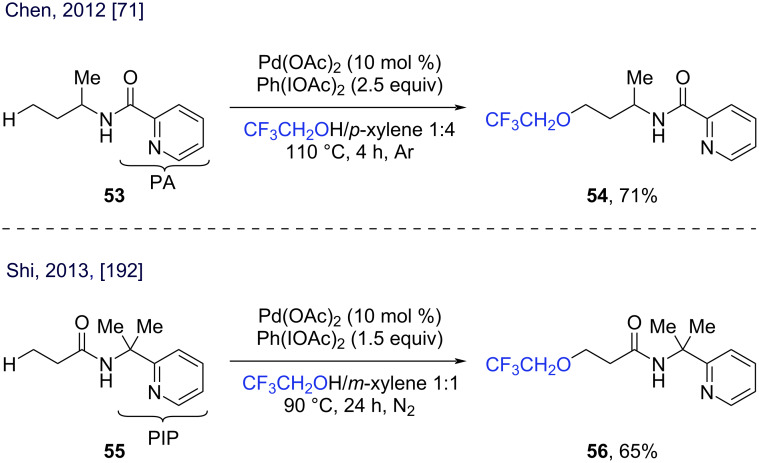
Pd(II)-catalyzed selective 2,2,2-trifluoroethoxylation of aliphatic amides using a bidentate directing group reported by the groups of Chen [71] and Shi [192].

The functionalization of C(sp^3^) centers by transition-metal-catalyzed C–H bond activation remains highly challenging [24,184–191]. In particular, the 2,2,2-trifluoroethoxylation of aliphatic derivatives is still limited to a handful of examples as illustrated by the two examples depicted in Scheme 24 [71,192]. Using a bidentate directing group (namely NHPA and CONHPIP for **53** and **55**, respectively), the groups of Chen [71] and Shi [192] independently reported the palladium-catalyzed selective 2,2,2-trifluoroethoxylation of aliphatic amines and amides at the γ and β positions, respectively, using trifluoroethanol as fluorination source in the presence of PIDA. Hence, an efficient access to the corresponding monoether derivatives **54** and **56** in 71% and 65%, respectively, were obtained.

## Conclusion

In summary, this review provides an overview of the major developments made over the last years for the synthesis of fluorinated compounds by transition-metal-catalyzed C–H bond activation. This review focused on the construction of C(sp^2^)–XR_F_ bonds and C(sp^3^)–XR_F_ bonds with an emphasis on the trifluoromethylation reaction and transformations using emergent fluorinated residues (SCF_3_, SeCF_3_, SCF_2_H, SCF_2_CO_2_Et or OCH_2_CF_3_ groups). Well-designed catalytic systems and suitable fluorinating sources were the key of success for these major developments.

Despite these advances, synthetic challenges still need to be overcome. These synthetic tools are so far still restricted to some fluorinated moieties and extension to other high value-added fluorinated residues [131,136,193–208] is of high importance. Besides, in comparison with the functionalization of C(sp^2^) centers on aromatic and vinylic derivatives, transition-metal-catalyzed functionalization of C(sp^3^)–H bonds remains largely underexplored to date. Furthermore, the development of enantioselective transformations allowing the synthesis of enantioenriched fluorine-containing compounds by transition-metal-catalyzed C–H bond activation will have a significant impact as for instance an access to pharmaceutically relevant derivatives. Finally, the use of abundant non-noble transition metals [209–211] in such reactions combined or not with modern technologies (photocatalysis and electrocatalysis) is still underexplored and any advances will be of high importance especially from a sustainability point of view aiming at developing greener synthetic routes towards fluorinated molecules.
